# POIBM: batch correction of heterogeneous RNA-seq datasets through latent sample matching

**DOI:** 10.1093/bioinformatics/btac124

**Published:** 2022-02-23

**Authors:** Susanna Holmström, Sampsa Hautaniemi, Antti Häkkinen

**Affiliations:** Research Program in Systems Oncology, Research Programs Unit, Faculty of Medicine, University of Helsinki, FI-00014 Helsinki, Finland; Research Program in Systems Oncology, Research Programs Unit, Faculty of Medicine, University of Helsinki, FI-00014 Helsinki, Finland; Research Program in Systems Oncology, Research Programs Unit, Faculty of Medicine, University of Helsinki, FI-00014 Helsinki, Finland

## Abstract

**Motivation:**

RNA sequencing and other high-throughput technologies are essential in understanding complex diseases, such as cancers, but are susceptible to technical factors manifesting as patterns in the measurements. These batch patterns hinder the discovery of biologically relevant patterns. Unbiased batch effect correction in heterogeneous populations currently requires special experimental designs or phenotypic labels, which are not readily available for patient samples in existing datasets.

**Results:**

We present POIBM, an RNA-seq batch correction method, which learns virtual reference samples directly from the data. We use a breast cancer cell line dataset to show that POIBM exceeds or matches the performance of previous methods, while being blind to the phenotypes. Further, we analyze The Cancer Genome Atlas RNA-seq data to show that batch effects plague many cancer types; POIBM effectively discovers the true replicates in stomach adenocarcinoma; and integrating the corrected data in endometrial carcinoma improves cancer subtyping.

**Availability and implementation:**

https://bitbucket.org/anthakki/poibm/ (archived at https://doi.org/10.5281/zenodo.6122436).

**Supplementary information:**

Supplementary data are available at *Bioinformatics* online.

## 1 Introduction

High-throughput molecular technologies are central to understanding complex diseases, such as cancers. However, datasets are often aggregated from several hospitals, treated with different protocols, and analyzed with different measurement technologies, which leads to unwanted factors creeping into the data, manifesting in experiment specific (‘batch’) patterns (Johnson *et al.*, 2007; Leek *et al.*, 2010). As the patterns in molecular profiles are directly used, e.g. discovering cancer subtypes and signatures (Levine *et al.*, 2013; The Cancer Genome Atlas Research Network, 2014, 2015, 2017), the batch effects can have vast implications on the interpretation and reproducibility of such studies. Overcoming the batch effect is particularly important in large data collections, such as The Cancer Genome Atlas (TCGA) (Weinstein *et al.*, 2013) and Genome Tissue Expression (Lonsdale *et al.*, 2013) projects, where the data originates from several hospitals and have varying data layers.

The impact of batch effects on the measurement data has been acknowledged (Johnson *et al.*, 2007; Leek *et al.*, 2010) and to some extent quantified (Buckley *et al.*, 2017; Lauss *et al.*, 2013; Rasnic *et al.*, 2019; Wang *et al.*, 2018). Still, standard practices resort to filtering (Levine *et al.*, 2013; The Cancer Genome Atlas Research Network, 2014, 2015, 2017) or statistical post-hoc analysis (The Cancer Genome Atlas Research Network, 2011). These, however, limit the statistical power and might bias the results. Batch effects can be mitigated using experimental design techniques (Katayama *et al.*, 2019). Unfortunately, this is only practical for specific, new experiments, and not for the large existing data collections or integration of different experiments. A complementary technique is to account for the batch effects computationally. For example, most differential expression callers, such as edgeR (Robinson *et al.*, 2010) and DESeq2 (Love *et al.*, 2014), can adjust the analysis for the presence of such factors. However, these models might be arduous to integrate into more complex analysis algorithms, such as network inference, and as a result, modular batch correction algorithms have been developed to preprocess the data for other analyses (Johnson *et al.*, 2007; Leek, 2014; Risso *et al.*, 2014; Zhang *et al.*, 2020).

A current limitation with all computational batch correction algorithms is that for the correction to be unbiased, both the batch labels and the experimental design factors must be known (Johnson *et al.*, 2007; Leek, 2014; Risso *et al.*, 2014; Zhang *et al.*, 2020). While this is possible for controlled experiments, it is impractical for patient derived samples, where the phenotypes are not known and still subject to the study. Moreover, many of the current methods are based on Gaussian models designed for microarray data, which are inherently biased for low-count sequencing data, characteristic e.g. to single-cell RNA-seq (Svensson *et al.*, 2018), and even at best impose a noise floor on the more sensitive molecular techniques negating their advantages. This is in contrast to e.g. differential expression methods, which exploit count models for improved sensitivity (Love *et al.*, 2014; Robinson *et al.*, 2010).

Here, we present a batch correction method POIsson Batch correction through sample Matching (POIBM), which is based on an idea of inferring virtual reference samples from the data. Consequently, special experimental designs or design factors are not required since POIBM automatically learns these from the data. This enables unbiased correction on complex patient data where the phenotypes are not known and exact replicates are not available. POIBM is designed to be optimal for RNA-seq count data, similar to ComBat-seq (Zhang *et al.*, 2020), which has been shown to outperform the Gaussian alternatives on RNA-seq data.

We use an engineered breast cancer cell line experiment (Rahman *et al.*, 2017) to show that POIBM exceeds the performance of previous methods, matching that of ComBat-seq (Zhang *et al.*, 2020), while being blind to the phenotypic labels unlike the other methods. Further, we use POIBM to correct the processing batch effects in the TCGA RNA-seq datasets across all cancers. We show that batch effects plague many cancer types; demonstrate that POIBM effectively discovers the existing replicates in stomach adenocarcinoma; and that integrating the POIBM-corrected data improves clinical subtyping in endometrial carcinoma.

## 2 Materials and methods

### 2.1 Modeling of RNA-seq data

Count data produced by a random process, such as stochastic gene expression (Raj and van Oudenaarden, 2008) or RNA-seq sampling, is expected to statistically follow Poisson distribution. In practice, the measurements exhibit Poisson-like distributions with more (Marioni *et al.*, 2008; McCarthy *et al.*, 2012, e.g. Poisson mixtures) or less (e.g. negative autoregulation, systematic artifacts) variance. These can be modeled either using a scaled Poisson (Hakkinen *et al.*, 2021) or a negative binomial distribution, the latter of which is suitable for the high variance case (e.g. mixtures of Poisson distributions). Neither is physically obvious, but for the increased flexibility for low variance data and the analytical simplicity we use the former (Hakkinen *et al.*, 2021).

In general, if $X_{ij}\in Z_{\geq 0}$ represent the read counts of an expression matrix, where the index $i\in Z_{\left[1,m\right]}$ runs over the *m* genes, and the index $j\in Z_{\left[1,n_{x}\right]}$ over the *n* samples, we consider a statistical model of a batch of RNA-seq data as follows:

(1) $$X_{ij}∼P(\lambda =c_{i\;}u_{ij\;}v_{j})$$

where P(λ) represents a Poisson distribution with the rate of $\lambda \in R_{\geq 0},\;c_{i}\in R_{\geq 0}$ are experiment-specific multiplicative batch coefficients (i.e. increased or reduced affinity for specific genes), $u_{ij}\in R_{\geq 0}$ are the underlying batch-free expression profiles, and $v_{j}\in R_{\geq 0}$ are total RNA factors (e.g. amount of sequenced material, amplification factors).

By definition, both the batch coefficients *c_i_* and the total RNA factors *v_j_* are independent of the sample and gene, respectively, and can be identified from a sufficiently sized dataset. Meanwhile, for the model to be identifiable, constrains on the payload model *u_ij_* must be imposed. Typically, either constant profiles (i.e. a rank-1 matrix factorization) or a low-rank known linear combination is used (Johnson *et al.*, 2007; Risso *et al.*, 2014; Zhang *et al.*, 2020), but a low-rank blind matrix factorization, as in RNA-seq decompositions (Hakkinen *et al.*, 2021), is possible.

### 2.2 Sample matching across batches

A challenge for batch correction is the inherent heterogeneity and lack of matching replicates between batches. We establish a mapping between each source sample and a virtual target sample, which is a probabilistic combination of the target samples. This way, the samples that map well will have a higher impact and for the ones that do not, the impact to the correction coefficient will average out, and a suitable ‘replicate’ can be interpolated instead of requiring an exact one to exist.

Let *X_ik_* be the read counts for the target and *Y_ij_* for the source experiment, as specified above. Following Equation (1), the batch model with virtual sample matching reads as follows:

(2) $$\begin{array}{c} X_{ik}∼P(c_{i}u_{ij}v_{x_{k}})\;\;\text{with probability}\;\;w_{kj}\\ Y_{ij}∼P(u_{ij}v_{y_{j}})\\ \;\text{such that}\;\;\frac{1}{n_{x}}\sum_{k=1}^{n_{x}}w_{kj}=1,\;\frac{1}{n_{y}}\sum_{j=1}^{n_{y}}w_{kj}=1, \end{array}$$

where *c_i_* represent the multiplicative batch coefficients from the source space to the target space (i.e. the batch correcting transformation); *u_ij_* are the expression profiles of the matching pairs; and $v_{x_{k}}$ and $v_{y_{j}}$ are the total RNA factors for the two datasets X and *Y*, respectively; as in above. Further, $w_{kj}\in [0,1]$ for $k\in Z_{\left[1,n_{x}\right]},\;j\in Z_{\left[1,n_{y}\right]}$ are the sample matching weights. The marginal convexity imposed on the matching weights keeps the sample matching from deteriorating into independent sets. Of note, the data could be also mapped into any common space, such as that of the geometric averages by substituting $u_{ij}\leftarrow {\sqrt{c_{i}}}^{-1}u_{ij}$.

When the data feature samples for which a matching sample cannot be interpolated, such as when the datasets are known to contain major unique phenotypes each, a trimming procedure is necessary: the model is imposed only on a fraction of samples *ρ_x_* and *ρ_y_* on the datasets X and *Y*, respectively. If *ρ_x_* and *ρ_y_* are chosen to be a lower bound of the fractions of shared phenotypes, the scheme remains unbiased.

### 2.3 Implementation and parameters

The model of Equation (2) can be optimized through multistage expectation maximization (EM). The details for scaled Poisson models are discussed in Hakkinen *et al.* (2021) and the derivation is given in the Supplementary Material. Specifically, the EM updates for the parameters are:

(3) $$\begin{array}{c} u_{ij}=\frac{\overset{}{\overset{︷}{{\left(\sum_{k=1}^{n_{x}}w_{kj}\right)}^{-1}\sum_{k=1}^{n_{x}}w_{kj}X_{ik}}\text{virtual}\text{target}\text{for}\;Y_{ij}}+Y_{ij}}{{\left(\sum_{k=1}^{n_{x}}w_{kj}\right)}^{-1}\sum_{k=1}^{n_{x}}w_{kj}c_{i}v_{x_{k}}+v_{y_{j}}}\\ v_{x_{k}}=\frac{\sum_{j=1}^{n_{y}}w_{kj}\sum_{i=1}^{m}X_{ik}}{\sum_{j=1}^{n_{y}}w_{kj}\sum_{i=1}^{m}c_{i}u_{ij}}\\ v_{y_{j}}{}^{-1}=\frac{\sum_{k=1}^{n_{x}}w_{kj}\sum_{i=1}^{m}X_{ik}}{\sum_{k=1}^{n_{x}}w_{kj}\sum_{i=1}^{m}c_{i}u_{ij}v_{x_{k}}v_{y_{j}}}={\left(\frac{\sum_{i=1}^{m}Y_{ij}}{\sum_{i=1}^{m}u_{ij}}\right)}^{-1}\\ c_{i}=\frac{\sum_{k=1}^{n_{x}}\sum_{j=1}^{n_{y}}w_{kj}X_{ik}}{\sum_{k=1}^{n_{x}}\sum_{j=1}^{n_{y}}w_{kj}u_{ij}v_{x_{k}}} \end{array}$$

which make the role of the virtual target as a weighted combination of the target samples obvious. The weights *w_kj_* are updated such that:

(4) $$\begin{array}{c} w_{kj}\propto \;\text{exp}\;\sum_{i=1}^{m}(X_{ik}\;\text{log}(c_{i}u_{ij}v_{x_{k}})-c_{i}\;u_{ij}\;v_{x_{k}}+\\ Y_{ij}\;\text{log}(u_{ij}v_{y_{j}})-u_{ij}v_{y_{j}}\\ -\;(X_{ik}\;\text{log}(X_{ik})-X_{ik}+\\ Y_{ij}\;\text{log}(Y_{ij})-Y_{ij})) \end{array}$$

and that the convexity constraints in Equation (2) are satisfied. If trimming is used, only the specified fraction of top values are set according to Equation (4), and the remaining *w_kj_* are set to zero. The updates of Equations (3) and (4) are iterated to convergence, which guarantees a local maximum of the likelihood of Equation (2).

The algorithm inputs the target read count matrix *X*, the source read count matrix *Y*, and some parameters: maximum number of EM iterations, number of restarts, and fraction of target *ρ_x_* and source samples *ρ_y_* to be used. The restarts are used to combat local solutions, which occur particularly when trimming is used. The restarts use uniform random initial assignment of the trimmed samples. The other values are initialized as *c_i_* = 1, $v_{x_{k}}\propto \sum_{i=1}^{m}X_{ik}$ and $v_{y_{j}}\propto \sum_{i=1}^{m}Y_{ij}$. We use 100 iterations, 20 restarts and $\rho_{x}=\rho_{y}=50\%$ unless otherwise mentioned.

The model directly provides the batch coefficients *c_i_*, and the total RNA factors $v_{x_{k}}$ and $v_{y_{j}}$ for each dataset. Moreover, the weights *w_kj_* hold information on to which target sample each source sample is being mapped. The batch correction from the source to the target space is finally applied by multiplying in the batch coefficients:

(5) $${\hat{Y}}_{ij}=c_{i}Y_{ij}$$

providing both datasets X and $\hat{Y}$ in the target space, but as the estimated batch coefficients *c_i_* are explicitly estimated, out-of-sample mapping of future data is also possible.

### 2.4 Breast cancer cell line data

A specially constructed RNA-seq experiment, as proposed in Zhang *et al.* (2020), was used for quantitative method comparison. The data consists of three batches of primary breast tissue that has been used to study breast cancer progression (McQuerry *et al.*, 2019; Rahman *et al.*, 2017). Each batch features case samples with an overexpression of a specific growth factor receptor network oncogene, induced by transfection. Each batch also contains control samples that have been transfected with a vector that expresses a green fluorescent protein. Batch 1 (GEO: GSE83083) has 5 samples overexpressing *HER2* with 12 controls; batch 2 (GEO: GSE59765) has 6 samples overexpressing *EGFR* with 6 controls; and batch 3 (GEO: GSE83083) has 9 samples overexpressing *KRAS* (G12V mutant) with 9 controls. Genes with zero expression in all samples of any batch were removed. The final matrix contained 47 samples and 18 013 genes. The samples can be divided into either three technical batches or four phenotypes, i.e. *HER2*, *EGFR*, *KRAS* or control.

After applying each method, the batch corrected data were scaled into counts per million (CPM) and log-transformed for comparison using principal component (PCA) and variance analysis (ANOVA) as in Zhang *et al.* (2020).

### 2.5 The Cancer Genome Atlas data

The Cancer Genome Atlas (TCGA) level 3 RNA-seq data for each cancer type was downloaded from the Broad GDAC Firehose portal (https://gdac.broadinstitute.org/) on March 3, 2021. Genes not present in all datasets were removed. In total, the data consisted of 59 datasets, 37 cancer types, 17 959 samples (some of which occurred in multiple datasets) and 21 184 genes. The data was split into 6 batches by the processing institute, pipeline and sequencing platform.

### 2.6 Comparison with previous methods

The alternative methods used for evaluation were original ComBat (Johnson *et al.*, 2007), ComBat-seq (Zhang *et al.*, 2020) (both of sva v3.38.0), RUVSeq v1.24.0 (Risso *et al.*, 2014) and PRISM v0.9.0-7 in rank-1 or linear factorization mode (Hakkinen *et al.*, 2021).

Like POIBM, each method assumes a multiplicative gene specific batch coefficients in the raw (count) RNA space. The methods differ in either the unmodeled variation (error model) or in the retained modeled variation (payload model). ComBat and RUVSeq use Gaussian error models in log-RNA space, while ComBat-seq uses a negative binomial error model and PRISM and POIBM use scaled Poisson models in raw RNA space. Specifically, the last three methods model explicitly discrete and heteroscedastic RNA-seq data. ComBat, ComBat-seq and RUVSeq allow a known linear payload (e.g. linear group factors), while more general methods like PRISM can further learn these factors (i.e. bilinear model), and POIBM uses probabilistic sample matching.

As for the input, ComBat requires the batch labels, the groups and a log-transformed count matrix. The output is a corrected matrix in the log-space. ComBat-seq uses the batch labels, groups and the count data matrix, while the parameter ‘shrink’ was set to false (default). The output is a corrected matrix in the raw count space. RUVSeq uses the groups and the count matrix, and the number of factors of unwanted variation *k* was set to 1. PRISM rank-1 factorization inputs one batch at a time and outputs the marginal gene and sample coefficients as in Equation (1), and the gene coefficient ratios were used for transformation as in Equation (5). PRISM in linear mode uses a design matrix encoding the batch and group labels. For POIBM and PRISM, the largest batch was used as the target space.

## 3 Results and discussion

### 3.1 POIBM: batch correction of heterogeneous RNA-seq samples through latent sample matching

POIBM is a novel method for correcting batch effects in RNA-seq data between heterogeneous populations. The novelty of the method is that two types of heterogeneity are tolerated: (i) distinct phenotypes in the samples composing the dataset—it is only necessary that a sufficient number of virtual target samples can be interpolated from a dataset; and (ii) stochasticity (noise) due to natural gene expression variability and RNA-seq sampling. Moreover, the underlying phenotypes need not to be known a priori, but are learned in the process, which means that special experimental design or replicate samples are not necessary.

**Fig. 1.**
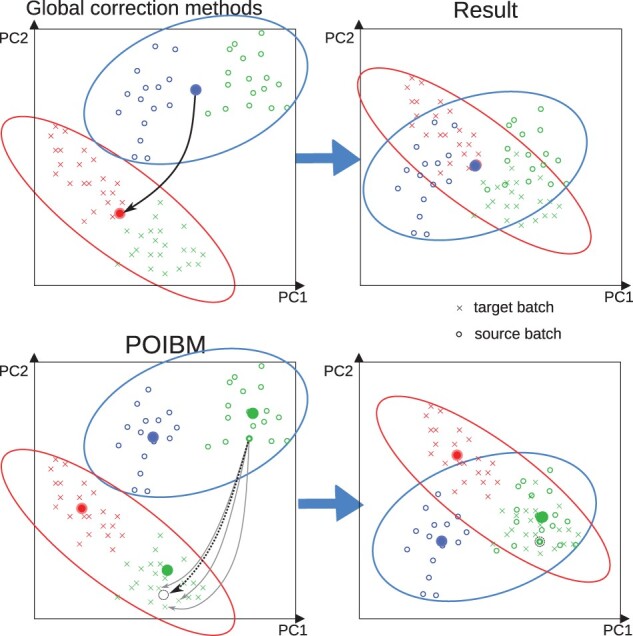
An overview of traditional batch correcting methods and POIBM. The data consists of two batches (*o*’s and *x*’s). There are two subpopulations within each batch (colors). The green subpopulations represent the same phenotype (e.g. controls), while the blue and red populations are batch specific (e.g. cases). The upper panels show that using the global population statistics (e.g. mean, solid circles in the figure) to find the correction coefficients overcorrects the shared control subpopulation and renders the unique subpopulations indistinguishable. The lower panels show how POIBM overcomes this by mapping each sample to a weighted virtual target sample, allowing the batch correction coefficient to be inferred only from the well matching subpopulations, which correctly harmonizes the shared subpopulations but leaves the unshared phenotypic differences intact

POIBM utilizes only two expression matrices of read counts, a target matrix and a source matrix. Each sample in the source matrix is compared with all samples in the target matrix, from which a virtual target sample is constructed (see Fig. 1 and Section 2). The batch effect is then inferred from the discrepancy between the source samples and their virtual targets—rather than the dataset averages—and the two steps are iterated to convergence. The procedure produces the batch correction coefficients for each gene, allowing mapping the source data to the target space; a weight matrix representing the sample matching; the total RNA factors for each sample; and the inferred underlying shared expression profiles, each of which can be output. Our implementation is freely available at https://bitbucket.org/anthakki/poibm/ under the simplified BSD license.

### 3.2 Quantitative comparison with previous methods

We performed a quantitative comparison with existing methods on a specially designed breast cancer cell line RNA-seq experiment that was published in Rahman *et al.* (2017) and used by Zhang *et al.* (2020) to evaluate different batch correction methods. These data consist of three batches with a shared control subpopulation mixed with a unique case subpopulation each (engineered to overexpress *HER2*, *EGFR* or *KRAS*), as detailed in Section 2. The methods used in the comparison were ComBat (Johnson *et al.*, 2007), ComBat-seq (Zhang *et al.*, 2020), RUVSeq (Risso *et al.*, 2014) and PRISM in rank-1 and linear modes (Hakkinen *et al.*, 2021), as detailed in Section 2.

We discovered that with a trimming factor of 50%, POIBM can perfectly identify the shared control samples (zero weight on the unshared samples), and infer the batch coefficients from these. This is unlike to the other methods, which require known batch labels, which were provided for the analysis. While running POIBM without trimming produced unsatisfactory results as the similar *EGFR* and control phenotypes tended to get intermixed, the exact choice of the trimming factor was not found to be important, and trimming to ∼30% to 60% produced qualitatively similar results (Supplementary Fig. S1). Similarly, the choice of the target space impacted accuracy but was not qualitatively important (Supplementary Fig. S1).

**Fig. 2.**
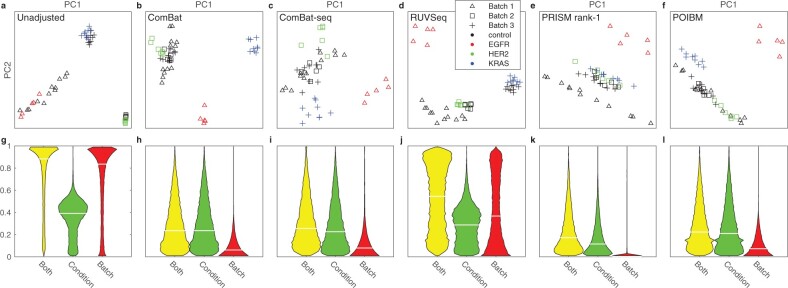
PCA projections and fraction of explained variance of the uncorrected and batch corrected data using various methods in the breast cancer cell line dataset, the different colors encoding batches and markers phenotypes. (**a–f**) Data projected to first two principal components (PC). (a) Unadjusted, (b) ComBat, (c) ComBat-seq, (d) RUVSeq, (e) PRISM rank-1, (f) POIBM. (**g–l**) Kernel density estimates (box kernel with a bandwidth of 0.02) of fraction of variance explained by the phenotypic variation (Condition), batch (Batch), or both (Both), over all the 18 013, with the six methods, respectively. While lines denote medians. The averages are tabulated in Table 1

Our results indicate that POIBM is effective in removing the unwanted batch variance, while the biological variance is left intact (Fig. 2f and l). The principal component analysis (PCA) projections of the corrected data (Fig. 2a–f) indicate that after batch correction the control samples of each batch cluster together, although the *EGFR* cases remain mixed with the control samples. However, the latter is exhibited by all of the methods, with and without both the batch and phenotypic information, suggesting that the phenotypes are in fact partly overlapping.

Quantitatively, POIBM retains a similar level of phenotypic (condition) variance as ComBat-seq (Fig. 2b and h) and ComBat (Fig. 2a and g), while being equally effective in removing batch-related variance (Table 1), as quantified by a set of one-tailed Bartlett’s test for equal variances. Meanwhile, RUVSeq appears (Fig. 2d and j) to be poor in removing the batch-related variance altogether, possibly disturbed by the correlation between the condition and batch labels, as it appears to be performed well in retaining the phenotypic variance. As an opposite reference, the PRISM rank-1 correction (Fig. 2e and k) is the most effective in removing batch variance but results in both the condition as well as the batch-specific variance (cf. Fig. 1) to be eliminated. POIBM performs most similar to PRISM in linear mode, which is expected on these data. The runtimes are tabulated in Supplementary Table S1.

**Table 1.** Fraction of variance in the data explained by batch, condition, or a combination of both after applying the different correction methods

Method	Both	Condition	Batch
Unadjusted	74.0%	35.6%	69.0%
ComBat	28.5% (*)	28.5% (x)	8.51% (*)
ComBat-seq	30.5% (*)	27.5% (x)	12.1% (*)
RUVSeq	52.8%	28.7% (x)	42.0%
PRISM rank-1	22.8% (*)	16.5% (**)	1.68% (**)
PRISM linear	27.3% (*)	26.4% (x)	8.82% (*)
POIBM	27.3% (*)	26.3% (x)	9.96% (*)

*Note*: The markers after the numbers indicate a significant difference (*P *<* *0.05, Bartlett’s test) between the methods: (*) significant difference from the unadjusted, no differences within the cases; (x) no differences within the cases; (**) significant difference from the unadjusted and all the others.

The results suggest that POIBM can significantly outperform both RUVSeq and the PRISM rank-1 on these data, and match the performance of the newest state-of-the-art methods such as ComBat-seq, with respect to both removing batch specific variation and retaining phenotypic variance. However, the main difference here is that ComBat-seq, ComBat, RUVSeq and linear PRISM are informed of which samples are controls and which are not (known, or non-blind phenotypic factors), while POIBM discovers the phenotypic labeling automatically (unknown, or blind phenotypic factors). Despite this lack of information, POIBM still performs no worse. Compared to rank-1 PRISM, which uses the same RNA-seq model than POIBM but lacks the phenotypic modeling, we conclude that the novel sample matching approach is the key to the superior performance.

The methods were also compared using Monte Carlo simulations as detailed in the Supplementary Material and summarized in Supplementary Figure S2.

### 3.3 Batch correction of TCGA data

#### 3.3.1 Processing batches in TCGA data

The Cancer Genome Atlas (TCGA) is a vast collection of molecular data from various cancer types. Despite the data being collected from various sources and analyzed at various institutions, studies of its batch effects are scarce. In most studies, the analysis is limited into a particular subset of samples to mitigate batch effects (Levine *et al.*, 2013; The Cancer Genome Atlas Research Network, 2014, 2015, 2017), or outright ignored.

**Fig. 3.**
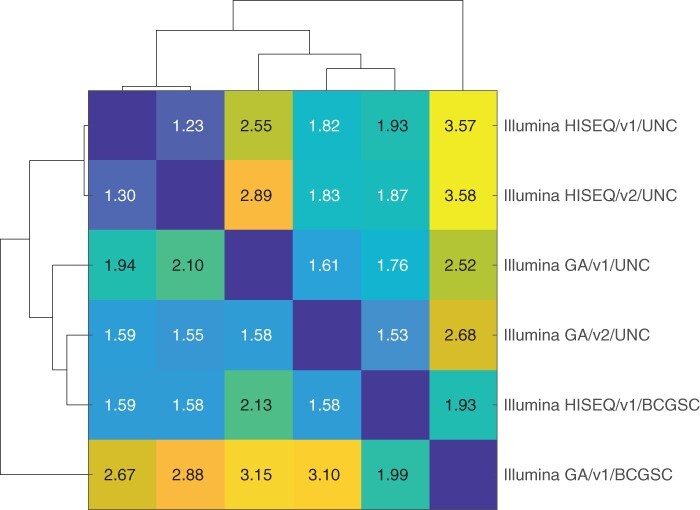
Processing batches and their dissimilarity in TCGA data across all the cancers. Directed dissimilarity between the processing batches of TCGA data, as estimated from the magnitude of variation of the batch correction factors using POIBM, along with their hierarchical clustering. Numbers reflect mean batch coefficient fold-change

On the TCGA level 3 expression data, one obvious source of batch effects is that the data are sequenced using either Illumina GA (‘illuminaga’) or HISEQ (‘illuminahiseq’) sequencing system, were processed using an older (‘rnaseq’, v1) or a newer pipeline (‘rnaseqv2’, v2), and were processed either at University of North Carolina (‘unc.edu’) or at Canada’s Michael Smith Genome Sciences Centre (‘bcgsc.ca’). These combinations yielded a total of six batches, a shown in Figure 3.

We performed a pairwise estimation of the batch effects in each batch-pair using POIBM, which quantifies the extent of batch effects between the different batches. Correction coefficient of unity is indicative of a lack of batch effect, while large deviations suggest more drastic batch effects. The deviation of the logarithmic batch coefficients from unity over all genes were consequently used as distances between the batches in a hierarchical clustering. The differences between the clusters (Fig. 3) indicate that the processing pipeline has have the least effect of the three, while both the sequencing platform and the processing institute exhibit larger differences. This is in line with previous findings that suggest that experimental factors rather than computational algorithms are the major source of batch induced bias (Rasnic *et al.*, 2019; Wang *et al.*, 2018). Still, the batch effect between any set is no less than ∼1.23× on average (cf. Fig. 3), suggesting that batch correction is necessary for accurate expression quantification.

By cancer type (Supplementary Fig. S3) the implications of these batch effects were discovered to group as follows: (i) 17 cancer types only feature data from a single batch. For these, the batch correction might be less urgent, unless the study includes integrative analysis with other collections or across the cancer types; (ii) Data have been processed with a newer pipeline (v2) but contain also the v1 data: BLCA, BRCA, HNSC, KIRP, LIHC, LUAD and THCA. For these, correcting the batch effects might be appealing, as several of the original TCGA reports were conducted with the older, reduced dataset (Levine *et al.*, 2013; The Cancer Genome Atlas Research Network, 2014, 2015, 2017). Also, LUSC is similar in that there are less samples in the newer batch, and for ESCA and OV the data are processed at a different institutions; (iii) Data from multiple batches with high overlap between the datasets: LAML and STAD. (iv) Data from multiple batches, but with a small overlap: COAD, READ and UCEC. In this group, the need for batch correction is more urgent, as combining the datasets can provide up to ∼67.6% more samples, increasing the analytical power.

#### 3.3.2 Replicate discovery in stomach adenocarcinoma

We inspected the POIBM established sample matchings in stomach adenocarcinoma (STAD) (The Cancer Genome Atlas Research Network, 2014). This cancer type is exemplary in the sense that the TCGA data features samples from three different batches: Illumina GA/rnaseq v1/BCGSC (36 samples), HISEQ/v1/BCGSC (271 samples) and HISEQ/v2/UNC (450 samples), with a large overlap between the samples of ∼67.1%. This makes it a good candidate in evaluating whether similar cancer patient samples can be automatically discovered by POIBM.

Between the clusters, we found that ∼95.7% of the mapping weight (∼291 samples) of the intersecting samples (304 samples) is indeed on the matching samples, which suggests that POIBM is capable in automatically identifying the replicates across the batches. The average mapping entropy for the intersecting source samples is ∼1.23 target samples, while for the non-intersecting source samples is ∼264 target samples, indicating that the virtual targets for the intersecting samples are mapped nearly one-to-one, while the non-intersecting samples are mapped nearly uniformly to the whole target population (cf. Fig. 1), as expected in the case of a mixed population of replicates and non-replicates. As a result, the batch coefficients are correctly inferred weighting in mostly the replicates (∼216× weight), as one would do in a perfectly informed approach.

Similar findings were made regarding the TCGA acute myeloid leukemia (LAML) data, as detailed in the Supplementary Material.

#### 3.3.3 Integration of uterine corpus endometrial carcinoma expression subtypes across batches

Next, we analyzed the data for uterine corpus endometrial carcinoma (UCEC) (Levine *et al.*, 2013). The TCGA data for UCEC features two batches: Illumina GA/v2/UNC (381 samples), HISEQ/v2/UNC (201 samples), and an overlap of 1 sample (∼0.262%). Again, these two clusters are quite distant in the overall batch effect variation (Fig. 3).

**Fig. 4.**
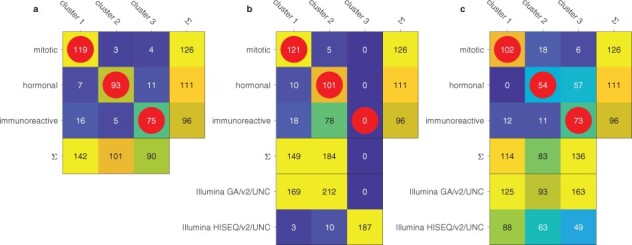
Reclustering of the TCGA UCEC samples. The classes ‘mitotic’, ‘hormonal’ and ‘immunoreactive’ represent those from Levine *et al.* (2013), while the clusters on the columns our reproduction. (**a**) Our best effort of reproducing the Levine *et al.*’s (2013) clustering, using only the same samples that were used in the study. (**b**) Clustering of all available TCGA UCEC dataset samples, across both batches, with no batch correction. (**c**) Clustering of all TCGA UCEC samples, with batch effects corrected using POIBM. The rows and columns with Σ represent marginal sums of the original 333 samples, and the ones named after the batches all samples in each. Red circles indicate best matching clusters

**Fig. 5.**
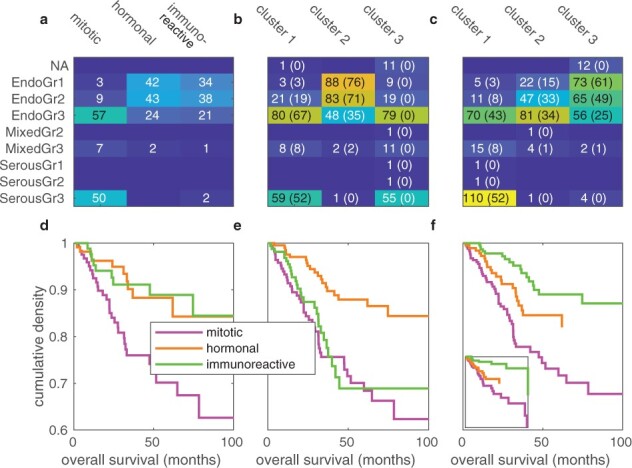
Clinical features of the TCGA UCEC expression clusters. Tumor histology and grade by the clusters: (**a**) for the 333 samples as in Levine *et al.* (2013), (**b**) for clustering all TCGA UCEC samples without batch correction and (**c**) for clustering all TCGA UCEC samples with batch effects corrected using POIBM. The numbers in parentheses indicate samples of the 333 original samples. Overall survival curves for the samples stratified by the clustering: (**d**) for the 333 samples as in Levine *et al.* (2013), (**e**) for clustering all samples without batch correction and (**f**) for clustering all samples with batch effects corrected with POIBM, with the inset showing the survival curves for the 333 original samples

Previously, Levine *et al.*, (2013) performed a clustering of 333 of the samples, reporting three expression subtypes dubbed as ‘mitotic’, ‘hormonal’ and ‘immunoreactive’. The samples in this study were from the Illumina GA/v2/UNC dataset, and presumably not all the data were available at the time of the analysis. We first verified that we can reproduce the clustering in Levine *et al.*, (2013). We were able to capture both the published labeling (Fig. 4a) and the histological features (Fig. 5a) as described in the work, though slight differences remained.

Next, we performed clustering using all the UCEC samples available in TCGA, including new samples and samples in the newer batch, for a total of 581 (versus 333 samples). We performed the clustering for both uncorrected data (Fig. 4b), which is expected to be susceptible to batch effects, and for batch corrected data using POIBM (Fig. 4c). Both the clustering on the original 333 samples and on the corrected data are able to well recover each of the clusters (*P*-values of $2.99\times 10^{-19}$ and $5.97\times 10^{-4}$ in a one-tailed multinomial test for diagonal enrichment). Meanwhile, clustering the uncorrected data was unable to recover the clustering of Levine *et al.*, (2013), neither with three (Fig. 4b; *P*-value 1) nor with four clusters (Supplementary Fig. S4a; *P*-value 1), but had suitable overlap with five clusters (Supplementary Fig. S4b; *P*-value of $3.54\times 10^{-20}$). This indicates that the clustering in Levine *et al.*, (2013) can be reproduced with the full dataset, but only when batch corrected, unless a much higher number of clusters, essentially modeling each batch and cluster combination, are used.

We found that the clusterings of the uncorrected data exhibit strong batch specificity (*P*-values >0.96 for specificity of 95% or more in a one-tailed binomial test, or equivalently specificity of 99.6% or more at a significance level of 0.05). This does not occur in the batch corrected clustering (*P*-value of $1.94\times 10^{-19}$, or significance for specificity of 81% or more). This suggests that the batch corrected values integrate well across the batches. We also found that in each case the batches correlate with the clustering (*P*-values $<5.74\times 10^{-5}$ in a two-tailed Fisher’s exact test), which suggests that a batch correction method like POIBM, which models both the batch and phenotypic effects simultaneously, is necessary to correct these data, as the populations are not equal in distribution. Also, we found that for each case the marginals, i.e. the cluster abundances, change significantly (*P*-values $<3.1\times 10^{-4}$ in a two-tailed multinomial test for equal distributions), except for the clustering of the original samples (Fig. 4a; *P*-value of 0.45), further evidencing that the addition of the new samples changes the overall population distribution.

With the new clustering, we examined whether the clinical properties of the clusters remain comparable to those reported in Levine *et al.*, (2013). First, we tested the specificity of the serous and grade 3 endometriod histologies to the mitotic cluster (Fig. 5a–c). In the original data, both of these significantly associate (*P*-values of $1.02\times 10^{-12}$ and $2.2\times 10^{-4}$ for the serous and endometriod grade 3, respectively, in a one-tailed Fisher’s exact test for enrichment), while in the uncorrected data they do not (*P*-values of 0.25 and 0.24). The corrected data exhibits association between the serous histology and the mitotic cluster (*P*-value of $3.6\times 10^{-33}$), but the endometriod grade 3 samples no longer solely associate with the mitotic cluster alone (*P*-value of 0.09; Fig. 5c).

We further examined the association of all the histological types and the grades with each of the three clustering (Fig. 5a–c). We found that all clusterings are significantly associated with the histological features (*P*-values $<3.37\times 10^{-33}$ in two-tailed Fisher’s exact test), but the association is weakest for the uncorrected clustering (odds ratio of 1.69) and strongest for the batch corrected clustering (odds-ratio of 1.74). The odds ratio for the original clustering sits in the middle at 1.70, indicating that besides the increased significance due to increased number of samples, the new data provide more accurate extraction of clinically associated clusters. Similarly, the clusterings of the original and the batch corrected data correlate with the mutational clusters (*P*-values $<5.3\times 10^{-4}$ in one-tailed multinomial test for enrichment) and the CNA clusters (*P*-values $<8.5\times 10^{-5}$) reported by Levine *et al.*, (2013), while the clustering of the uncorrected data does not (*P*-values >0.42).

We tested the association with between the clusters and patient overall survival (Fig. 5d–e). The survival curves stratified by the clustering significantly differ in all cases (*P*-values of $5.2\times 10^{-4},\;6.4\times 10^{-57}$ and $2.8\times 10^{-68}$ for original, uncorrected and corrected, respectively, in a two-tailed 3-way log-rank test). However, only for the corrected clustering are all two-way comparisons significant (*P*-values <0.03), whereas for the original (*P*-value of 0.95) and the uncorrected (*P*-value of 0.21) the mitotic and the hormonal clusters, respectively, significantly differ from the other two which are not significant. This further supports the finding that adding the new, batch corrected data does not only yield more statistical power but also a clinically more refined picture of the two minor clusters.

We note that the uterine serous tumors share genomic features with serous ovarian and basal-like breast cancers (Levine *et al.*, 2013), so it is plausible that the immunoreactive expression subtype has been overlooked in the original data with respect to the patient overall survival, as clinically associated immunoreactive subtypes have been observed in both ovarian (The Cancer Genome Atlas Research Network, 2011; Verhaak *et al.*, 2013) and breast cancers (Ciriello *et al.*, 2015). Consequently, our analysis of the full batch corrected expression dataset facilitates integration with the corresponding genomic data from TCGA, which might provide insights into the immunoreactive endometrial carcinomas as well.

## 4 Conclusion

We designed POIBM, an effective method for batch correction of RNA-seq data that is designed to perform well on heterogeneous populations and on discrete, noisy RNA-seq data. The uniqueness of the method is that for each source sample a virtual target sample is formed from the target dataset, from which the batch coefficients are inferred. This implies that the matching samples need not to be known, and replicates need not to exist, but these are learned from the data. POIBM also models the discrete and heteroscedastic nature of RNA-sequencing or other count data, which makes it suitable for low-coverage sequencing data.

We used engineered breast cancer cell line overexpression experiments to show that POIBM can automatically learn the underlying phenotypic structure and matching replicate samples. Furthermore, our results indicate that even in the absence of the phenotypic labels it performs at least as well as the existing methods, which require such labeling. This is essential for complex datasets like cancer patient samples where the phenotypes are not known a priori, but are under investigation from the datasets in question and/or feature variation even within the samples of the same tumor.

By examining the publicly available TCGA RNA-seq data, we discovered that many of the cancer types contain their data distributed over two or more technical batches, and we showed that these technical factors can disturb the downstream analyses of the data if used without correction. We harmonized all the 17 959 RNA-seq samples with the six batches across all the cancer types, which facilitates the use of TCGA expression data for more accurate analyses and for unbiased comparisons across cancers. Specifically, we showed that in stomach adenocarcinoma POIBM can accurately identify the technical replicates, which allows identification of the implications of the batch effects, while in endometrial carcinoma we showed that batch harmonization using POIBM is necessary to extract full clinical power from the expression dataset.

We expect our methodology is indispensable for analyses integrating data from various collections, and even for long-running data collections, as the sequencing platforms and analysis pipelines cannot necessary be kept fixed. Our approach generalizes directly to any count data such as proteomics, and can be influential in advancing batch correction efforts for other molecular data domains.

## Supplementary Material

btac124_Supplementary_Data
