# Supplementary material for: POIBM: batch correction of heterogeneous RNA-seq datasets through latent sample matching
Source: Bioinformatics. 2022 Feb 23;38(9):2474–80. doi: 10.1093/bioinformatics/btac124 (PMC9048693; doi:10.1093/bioinformatics/btac124)
Supplement: btac124_Supplementary_Data [file btac124_supplementary_data.pdf]

Supplementary material for "POIBM: Batch correction of heterogeneous RNA-seq datasets through latent sample matching"

Susanna Holmström, Sampsa Hautaniemi, and Antti Häkkinen

Model derivation

Modeling sequencing counts and probabilistic sample matching

Let $X_{ik} \in \mathbb{R}_{\geq 0}$ and $Y_{ij} \in \mathbb{R}_{\geq 0}$ denote the random variables corresponding to the target and source samples, respectively, the rows denoting the m genes and the columns to the n_x and n_y samples. We assume the data can be modeled using a Poisson distribution, with total RNA scale factors:

$$\begin{aligned} X_{ik} &\sim \mathcal{P}(\ u_{x_{ik}}\ \ v_{x_k}\ ) \\ Y_{ij} &\sim \mathcal{P}(\ u_{y_{ij}}\ \ v_{y_j}\ ) \end{aligned} \tag{S1}$$

where $\mathcal{P}(\lambda)$ denotes a Poisson distribution with the rate λ and $u_{x_{ik}} \in \mathbb{R}_{\geq 0}$ and $u_{y_{ij}} \in \mathbb{R}_{\geq 0}$ represent the expression profiles, and $v_{x_k} \in \mathbb{R}_{\geq 0}$ and $v_{y_j} \in \mathbb{R}_{\geq 0}$ are the total RNA factors.

When the samples are unrelated, no assumption of the underlying profiles u_{x_ik} and u_{y_j} are made. Meanwhile, when two samples $X_{:k}$ and $Y_{:j}$ are related, a relationship through multiplicative batch coefficients is assumed: $u_{x_{ik}} = c_i \, u_{y_{ij}}$ for some $c_i \in \mathbb{R}_{\geq 0}$. In POIBM, the matching samples are not known, but are modeled using using latent random flag variables $Z_{kj} \in \{0, 1\}$. An explicit formulation of the full generative state-conditional model is:

$$\left.\begin{aligned} X_{ik} &\sim \mathcal{P}(\ c_i\ u_{ij}\ \ v_{x_k}\ ) \\ Y_{ij} &\sim \mathcal{P}(\ \quad u_{ij}\ \ v_{y_j}\ ) \end{aligned}\right\} \quad \text{given } Z_{kj} = 1$$
$$\left.\begin{aligned} X_{ik} &\sim \mathcal{P}(\ \quad \lambda_{x_{ik}}\quad\ ) \\ Y_{ij} &\sim \mathcal{P}(\ \quad \lambda_{y_{ij}}\quad\ ) \end{aligned}\right\} \quad \text{given } Z_{kj} = 0 \tag{S2}$$

where Z_{kj} are latent matching flags, u_{ij} represents the expression profile in the source space (that of Y_{ij}), and $\lambda_{x_{ik}} = u_{x_{ik}} \, v_{x_k}$ and $\lambda_{y_{ij}} = u_{y_{ij}} \, v_{y_j}$ following the independent model of Equation (S1).

The full density of the model in Equation (S2) is:

$$
\begin{aligned}
&\mathbb{P}\Big[\big((X_{ik})_{i=1}^{m}\big)_{k=1}^{n_x},\ \big((Y_{ij})_{i=1}^{m}\big)_{j=1}^{n_y},\ \big((Z_{kj})_{k=1}^{n_x}\big)_{j=1}^{n_y} \Big] \\
&= \prod_{i=1}^{m}\prod_{k=1}^{n_x}\prod_{j=1}^{n_y} \Big(\mathbb{I}[Z_{kj}=1]\, f_{\mathcal{P}}(\, X_{ik} \,|\, \lambda = c_i\, u_{ij}\, v_{x_k}\,)\, f_{\mathcal{P}}(\, Y_{ij} \,|\, \lambda = u_{ij}\, v_{y_j}\,)\ \mathbb{P}[Z_{kj}=1] \ + \\
&\qquad \mathbb{I}[Z_{kj}=0]\, f_{\mathcal{P}}(\, X_{ik} \,|\, \lambda = \lambda_{x_{ik}}\,)\, f_{\mathcal{P}}(\, Y_{ij} \,|\, \lambda = \lambda_{y_{ij}}\,)\ \mathbb{P}[Z_{kj}=0] \Big)
\end{aligned} \tag{S3}
$$

where $f_{\mathcal{P}}(\, x \,|\, \lambda) = \frac{\lambda^x}{x!}\exp(-\lambda)$ is the Poisson density, and $x!$ is the factorial of x.

As the absolute degree of matching is not of our immediate interest, we substitute for normalized matching variables $\bar{Z}_{kj} \doteq Z_{kj} \,|\, (\sum_{k=1}^{n_x}\sum_{j=1}^{n_y} Z_{kj} = 1)$, giving:

$$
\begin{aligned}
&\mathbb{P}[\, (X_{ik})_{i=1}^{m}, (Y_{ij})_{i=1}^{m}, \bar{Z}_{kj} = 1\,] \\
&= \prod_{i=1}^{m} \overbrace{\frac{\mathbb{P}[X_{ik}, Y_{ij} | Z_{kj}=1]}{\mathbb{P}[X_{ik}, Y_{ij} | Z_{kj}=0]}}^{\text{data likelihood ratio}} \ \overbrace{\frac{\mathbb{P}[Z_{kj}=1]}{\mathbb{P}[Z_{kj}=0]}}^{\text{prior ratio}} \ \overbrace{\prod_{k'=1}^{n_x}\prod_{j'=1}^{n_y} \mathbb{P}[X_{ik'}, Y_{ij'} | Z_{k'j'}=0]\ \mathbb{P}[Z_{k'j'}=0]}^{\text{constant with respect to } k,\ j} \\
&\propto \prod_{i=1}^{m} \frac{f_{\mathcal{P}}(\, X_{ik} \,|\, \lambda = c_i\, u_{ij}\, v_{x_k}\,)}{f_{\mathcal{P}}(\, X_{ik} \,|\, \lambda = \lambda_{x_{ik}}\,)} \frac{f_{\mathcal{P}}(\, Y_{ij} \,|\, \lambda = u_{ij}\, v_{y_j}\,)}{f_{\mathcal{P}}(\, Y_{ij} \,|\, \lambda = \lambda_{y_{ij}}\,)} \ \doteq \ \prod_{i=1}^{m} \Lambda_{ikj} \ \dot{\propto} \ w_{kj}
\end{aligned} \tag{S4}
$$

which are likelihood ratios Λ_{ikj} for the matching models versus the model of unrelated samples, and whose aggregate over the genes with an appropriate scaling is denoted by the weights w_{kj}. Note that these give convenient access to the conditional models $\mathbb{P}[\, \bar{Z}_{kj} \,|\, (X_{ik})_{i=1}^{m}, (Y_{ij})_{i=1}^{m}\,] = w_{kj} \,/\, \sum_{k=1}^{n_x}\sum_{j=1}^{n_y} w_{kj}$ and $\mathbb{P}[\, X_{ik}, Y_{ij} \,|\, \bar{Z}_{kj}\,] = \Lambda_{ikj} \,/\, \sum_{X_{ik}=0}^{\infty}\sum_{Y_{ij}=0}^{\infty} \Lambda_{ikj}$. In the above, the prior ratios $\mathbb{P}[Z_{kj}=1] \,/\, \mathbb{P}[Z_{kj}=0]$ are assumed to factor out, that is, each sample-pair is assumed the same prior susceptibility of being in the matching set, but a prior weight matrix could simply be factored into w_{kj} for a more general form.

Parameter estimation

The direct maximization of Equation (S3) is challenging due its mixture structure, but is very amenable to approximative iteration, such as the expectation maximization (EM) algorithm (Dempster *et al.*, 1977). Expectation maximization involves successively optimizing $\mathbb{E}_{\mathbf{Z}|\mathbf{X},\boldsymbol{\phi}}[\log \mathbb{P}[\, \mathbf{X} \,|\, \mathbf{Z}, \boldsymbol{\theta}\,]$ for $\boldsymbol{\theta}$, where $\mathbf{X}$ and $\mathbf{Z}$ are the observed and latent variables, respectively, and $\boldsymbol{\phi}$ are the current parameters, giving a local maximum of the observed data $\mathbb{P}[\, \mathbf{X} \,|\, \boldsymbol{\theta}\,] = \int_{\mathbf{Z}} \mathbb{P}[\, \mathbf{X}, \mathbf{Z}\,]\, \partial\mathbf{Z}$.

Given the density of Equation (S4), the expected log-likelihood $\bar{\ell}$ given the latent distribution w_{kj} lacking the constant global scaling factor is:

$$
\begin{aligned}
\bar{\ell} \doteq \sum_{i=1}^{m}\sum_{k=1}^{n_x}\sum_{j=1}^{n_y} \Big(& w_{kj}\ \big(X_{ik}\ \log c_i\, u_{ij}\, v_{x_k} - c_i\, u_{ij}\, v_{x_k} - \log X_{ik}! + Y_{ij}\ \log u_{ij}\, v_{y_j} - u_{ij}\, v_{y_j} - \log Y_{ij}! \big) \\
& - w_{kj}\ \big(X_{ik}\ \log \lambda_{x_{ik}} - \lambda_{x_{ik}} - \log X_{ik}! + Y_{ij}\ \log \lambda_{y_{ij}} - \lambda_{y_{ij}} - \log Y_{ij}! \big) \Big)
\end{aligned} \tag{S5}
$$

whose partial derivatives are:

$$
\begin{aligned}
\nabla_{c_i}\,\bar{\ell} &= \sum_{j=1}^{n_y}\sum_{k=1}^{n_x} w_{kj}\,\left(\frac{X_{ik}}{c_i} - u_{ij}\,v_{x_k}\right) \quad , \quad \nabla_{u_{ij}}\,\bar{\ell} = \sum_{k=1}^{n_x} w_{kj}\,\left(\frac{X_{ik}}{u_{ij}} - c_i\,v_{x_k}\right) + \sum_{k=1}^{n_x} w_{kj}\,\left(\frac{Y_{ij}}{u_{ij}} - v_{y_j}\right) \\
\nabla_{v_{x_k}}\,\bar{\ell} &= \sum_{i=1}^{m}\sum_{j=1}^{n_y} w_{kj}\,\left(\frac{X_{ik}}{v_{x_k}} - c_i\,u_{ij}\right) \quad , \quad \nabla_{v_{y_j}}\,\bar{\ell} = \sum_{i=1}^{m}\sum_{k=1}^{n_x} w_{kj}\left(\frac{Y_{ij}}{v_{y_j}} - u_{ij}\right)
\end{aligned} \tag{S6}
$$

These induce the marginal invariants:

$$
\begin{aligned}
\sum_{k=1}^{n_x}\sum_{j=1}^{n_y} w_{kj}\,X_{ik} &= \sum_{k=1}^{n_x}\sum_{j=1}^{n_y} w_{kj}\,c_i\,u_{ij}\,v_{x_k} \\
\sum_{k=1}^{n_x} w_{kj}\,X_{ik} + \sum_{k=1}^{n_x} w_{kj}\,Y_{ij} &= \sum_{k=1}^{n_x} w_{kj}\,c_i\,u_{ij}\,v_{x_k} + \sum_{k=1}^{n_x} w_{kj}\,u_{ij}\,v_{y_j} \\
\sum_{i=1}^{m}\sum_{j=1}^{n_y} w_{kj}\,X_{ik} &= \sum_{i=1}^{m}\sum_{j=1}^{n_y} w_{kj}\,c_i\,u_{ij}\,v_{x_k} \\
\sum_{i=1}^{m}\sum_{k=1}^{n_x} w_{kj}\,X_{ik} &= \sum_{i=1}^{m}\sum_{k=1}^{n_x} w_{kj}\,c_i\,u_{ij}\,v_{x_k} \qquad \Leftrightarrow \qquad \sum_{i=1}^{m} Y_{ij} = \sum_{i=1}^{m} u_{ij}\,v_{y_j}
\end{aligned} \tag{S7}
$$

that is, the above weighted marginal averages of the data and their corresponding model are matched at the optimum. These justify why the global scale in w_{kj} can be neglected for inference. The gradient roots follow:

$$
\begin{aligned}
c_i &= \frac{\sum_{k=1}^{n_x}\sum_{j=1}^{n_y} w_{kj}\,X_{ik}}{\sum_{k=1}^{n_x}\sum_{j=1}^{n_y} w_{kj}\,u_{ij}\,v_{x_k}} \quad , \quad u_{ij} = \frac{\sum_{k=1}^{n_x} w_{kj}\,X_{ik} + \sum_{k=1}^{n_x} w_{kj}\,Y_{ij}}{\sum_{k=1}^{n_x} w_{kj}\,c_i\,v_{x_k} + \sum_{k=1}^{n_x} w_{kj}\,v_{y_j}} \\
v_{x_k} &= \frac{\sum_{i=1}^{m}\sum_{j=1}^{n_y} w_{kj}\,X_{ik}}{\sum_{i=1}^{m}\sum_{j=1}^{n_y} w_{kj}\,c_i\,u_{ij}} \quad , \quad v_{y_j} = \frac{\sum_{i=1}^{m} Y_{ij}}{\sum_{i=1}^{m} u_{ij}}
\end{aligned} \tag{S8}
$$

and, as expected, the maximum likelihood estimators following the same logic for the unrelated models are $\lambda_{x_{ik}} = X_{ik}$, $\lambda_{y_{ij}} = Y_{ij}$, which are needed for updating w_{kj}.

What remains is to determine the updated latent variable distribution $\mathbf{Z}\,|\,\mathbf{X}, \boldsymbol{\phi}$ through the Bayes theorem, as $\mathbb{P}[\,\mathbf{Z}\,|\,\mathbf{X}, \boldsymbol{\phi}\,] \propto \mathbb{P}[\mathbf{X}\,|\,\mathbf{Z}, \boldsymbol{\phi}]\ \mathbb{P}[\mathbf{Z}\,|\,\boldsymbol{\phi}]$.

As given in Equation (S4):

$$
\begin{aligned}
w_{kj} &\propto \prod_{i=1}^{m} \frac{f_{\mathcal{P}}(\,X_{ik}\,|\,\lambda = c_i\,u_{ij}\,v_{x_k}\,)}{f_{\mathcal{P}}(\,X_{ik}\,|\,\lambda = X_{ik}\,)}\,\frac{f_{\mathcal{P}}(\,Y_{ij}\,|\,\lambda = u_{ij}\,v_{y_j}\,)}{f_{\mathcal{P}}(\,Y_{ij}\,|\,\lambda = Y_{ij}\,)} \\
&= \exp\sum_{i=1}^{m}\Big(X_{ik}\,\log c_i\,u_{ij}\,v_{x_k} - c_i\,u_{ij}\,v_{x_k} + Y_{ij}\,\log u_{ij}\,v_{y_j} - u_{ij}\,v_{y_j} \\
&\qquad - (X_{ik}\,\log X_{ik} - X_{ik} + Y_{ij}\,\log Y_{ij} - Y_{ij})\Big)
\end{aligned} \tag{S9}
$$

The parameter estimation procedure can be summarized as:

1. Choose initial parameters c_i, u_{ij}, v_{x_k}, and v_{y_j} and the latent variable weights w_{kj}. We choose $c_i = 1$ (no batch effect), $v_{x_k} \propto \sum_{i=1}^{m} X_{ik}$, $v_{y_j} \propto \sum_{i=1}^{m} Y_{ij}$ (total RNA factors proportional to the observed abundance), $w_{kj} \propto 1$ (uniform sample matching), and u_{ij} are initialized through Equation (S8).
2. Update parameters according to Equation (S8). As the updates are mutually recursive, a schedule is needed. We first update u_{ij}, then v_{x_k} and v_{y_j}, and finally c_i.
3. Update the latent variable distribution through Equation (S9).
4. Go to step 2 unless the iterates are converged, as indicated by the gradient norm of Equation (S5).

Constraining sample matching

Without any constraints on Z_{kj}, the solution to the model of Equation (S2) is trivial. Namely, the most likely case is the expression differences are explained by different expression profiles (the $Z_{kj} = 0$ branch of the model), as this allows most flexibility and the model to match the data perfectly.

In the above, this problem is circumvented by the usage of normalized latent variables $\bar{Z}_{kj}$ instead, which suggest a corresponding normalization on w_{kj}. Other choices are possible, such as normalizing for equal posterior source contributions, by letting $\sum_{k=1}^{n_x} w_{kj} \propto 1$; or normalizing for equal posterior target contributions, by letting $\sum_{j=1}^{n_y} w_{kj} \propto 1$. We found that the best strategy is to combine both. A scaling that satisfies both constraints simultaneously is:

$$\frac{1}{n_x} \sum_{k=1}^{n_x} w_{kj} = 1 \quad , \quad \frac{1}{n_y} \sum_{j=1}^{n_y} w_{kj} = 1 \tag{S10}$$

The intuition behind enforcing these constraints is that, likewise, for any single source *and* any single target sample, a set of batch coefficients c_i exist that fit the data perfectly, which tends to attract solutions locally in the parameter optimization. Similarly, for a single source *or* a single target, the optimization can drift to areas where c_i are overfit to a specific sample, stalling the optimizer. More generally, this limits the operation of any batch inference procedure where both the batch coefficients c_i and the phenotypes u_{ij} are unknown and the batches consist of single samples.

Practically, the following iterative algorithm can be used to combine Equation (S9) and Equation (S10):

1. Compute w_{kj} according to Equation (S9)
2. Row-normalize the values: $w_{kj} \leftarrow w_{kj} / \frac{1}{n_y} \sum_{j=1}^{n_y} w_{kj}$
3. Column-normalize the values: $w_{kj} \leftarrow w_{kj} / \frac{1}{n_x} \sum_{k=1}^{n_x} w_{kj}$
4. Go to step 2, unless w_{kj} have converged

Sample trimming

Further, when the datasets contain unique subpopulations, which cannot be interpolated to match without causing bias, a sample trimming procedure might be necessary. Our trimming procedure is as follows:

1. Compute w_{kj} according to Equation (S9)
2. Pick an active subset $K \times J \subset \{1, \cdots, n_x\} \times \{1, \cdots, n_y\}$ of given dimensions $|K| = \rho_x \, n_x$, $|J| = \rho_y \, n_y$ of w_{kj} containing its top elements, and zero the remaining elements
3. Apply normalization as in Equation (S10) on the selected active set (K, J)

The trimming is controlled by the trimming factors ρ_x and ρ_y, which specify the fraction of samples from the target and source spaces, respectively, from which the batch coefficients are inferred. On one hand, if the trimming factors are too large, bias can be induced in the batch coefficient estimates, and on the other, if the trimming factors are too small, POIBM suffers from increased estimator variance and, at the extreme of the range, putatively from overfitting. Practical trimming factors range from 25% to 75% (cf. Figure S1, Figure S2), a higher value balancing increased accuracy at the risk of bias on dissimilar populations. Note that trimming is not necessary if the populations of X, Y cover the same state space but have unequal densities, as the variable weights allow sample interpolation, so POIBM even with $\rho = 1$ is strictly more flexible than a non-phenotype aware model (cf. Figure S2).

Quantitative evaluation on Monte Carlo simulations

To determine the accuracy of batch coefficient and expression profile estimation, we used a series of Monte Carlo simulations with data generated as follows:

$$X_{ij} \sim \mathcal{P}(\lambda = d \, C_{i,K_j} \, U_{i,L_j} \, v_j) \tag{S11}$$

where $\mathcal{P}(\lambda)$ represents a Poisson distribution with the rate λ, i runs over the m genes, j runs over the n samples, d is the average per gene sequencing depth, $C_{i,k}$ represents the batch coefficient of the i:th gene for the k:th batch, $U_{i,l}$ represents the normalized expression of the i:th gene for the l:th phenotype, v_j represents the normalized total RNA factor the sample j, X_{ij} represents the observed counts at the i:th gene for the j:th sample, and K_j and L_j represent the batch and the phenotype labels of the j:th sample (discussed further below).

To avoid presenting any other structure in the data for the methods to exploit than those described by the batch and phenotype labels, $C_{i,k}$, $U_{i,l}$ and v_j are generated from maximum entropy distributions, namely:

$$\begin{aligned} \frac{1}{m} C_{:,k} &\sim \mathcal{D}(\boldsymbol{\alpha} = (1)_{i=1}^m) \\ \frac{1}{m} U_{:,l} &\sim \mathcal{D}(\boldsymbol{\alpha} = (1)_{i=1}^m) \\ \frac{1}{n} v_j &\sim \mathcal{D}(\boldsymbol{\alpha} = (1)_{i=1}^n) \end{aligned} \tag{S12}$$

where $\mathcal{D}(\boldsymbol{\alpha})$ represents a k-dimensional Dirichlet distribution with the precision parameters $\boldsymbol{\alpha} = (\alpha_i)_{i=1}^{k}$, $\boldsymbol{\alpha} = \mathbf{1}$ yielding vectors in the k-dimensional unit simplex with uniform density in the $k-1$ dimensional space. The scaling factors are used to scale each element to average unity. These problems are likely harder than a real world data, such as that presented in the manuscript, but serve a fair setting for evaluating the qualitative behavior.

For simplicity, we used two batches $k \in \{1, 2\}$, $k = 1$ representing a target and $k = 2$ a source batch, with three different phenotypes $l \in \{0, 1, 2\}$, $l = 0$ representing the shared, and $l = 1$ and $l = 2$ the unique phenotypes for the batches $k = 1$ and $k = 2$, respectively. The choice of numbering is arbitrary as the two batches have symmetric statistical properties. The parameter $\alpha \doteq \frac{1}{n}\sum_{j=1}\mathbb{I}[L_j = 0]$ represents the fraction of shared phenotype samples, and $\beta \doteq \frac{1}{n}\sum_{j=1}\mathbb{I}[K_j = 1]$ the fraction of target batch samples, which control asymmetries of the datasets.

The true target-over-source batch coefficient c_i and the expression ratio (fold-change) d_i implied the above model are:

$$\begin{aligned} c_i &\doteq C_{i,1} \,/\, C_{i,2} \\ d_i &\doteq U_{i,1} \,/\, U_{i,2} \end{aligned} \tag{S13}$$

which are compared with the estimates by the different methods. Specifically, geometric standard deviation (i.e. average logarithmic fold-change deviation) between the true value and the estimate was quantified by using a robust (scaled mean absolute deviation) estimator. If a method does not naturally provide a corresponding estimate, one can be derived from the uncorrected X_{ij} and the batch corrected data $\hat{X}_{ij}$:

$$\begin{aligned} \hat{c}_i &\doteq \left(\sum_{j=1}^{n}\mathbb{I}[K_j = 2]\,\hat{X}_{ij}\right) \,/\, \left(\sum_{j=1}^{n}\mathbb{I}[K_j = 1]\,X_{ij}\right) \\ \hat{d}_i &\doteq \left(\sum_{j=1}^{n}\mathbb{I}[L_j = 2]\,\hat{X}_{ij}\right) \,/\, \left(\sum_{j=1}^{n}\mathbb{I}[L_j = 1]\,\hat{X}_{ij}\right) \end{aligned} \tag{S14}$$

which are the Poisson maximum likelihood estimates given X_{ij} and $\hat{X}_{ij}$.

The default parameters used were $m = 10{,}000$ genes, $n = 100$ samples, average sequencing depth $d = 100$, shared-unshared bias of $\alpha = 0.5$, and target-source bias of $\beta = 0.5$. The parameters d, n, α, and β were each varied independently (cf. Figure S2), giving a total of 55 different settings. The time of performing batch correction for each of the 55 was recorded for each method, not including library loading, Monte Carlo simulation, or result evaluation time. A total of 100 replicate simulations were run for each setting. We were unable to make RUVSeq to run on these data reliably, likely due it relying on identification of non-differentially expressed control genes (Risso *et al.*, 2014).

The error in the batch coefficient and expression ratio estimates are shown in Figure S2. The results indicate that POIBM works well except for very low global sequencing depths (e.g. $\ll 10$) and when there are very few samples in the shared population (e.g. $\ll 5$), either due to lack of samples in general or due to

the datasets being unbalanced toward having mostly unique samples. Generally, the methods with known phenotypes have smaller estimator variance, but the performance is qualitatively similar for POIBM. For balanced datasets (i.e. $\alpha \approx 0.5$, $\beta \approx 0.5$) POIBM can outperform both ComBat and ComBat-seq, likely due to a more optimal model specific to this simulation, but not the non-blind method with same model (PRISM linear). Using POIBM with no trimming ($\rho = 1$) can be problematic, unless the common phenotype fraction is above ∼66%. However, the performance is better than that of the phenotype naive methods (PRISM rank-1). Meanwhile, POIBM with a small trimming factor is advantageous for low number of shared phenotypes (below ∼33%), but otherwise performs similarly to the larger trimming factors except for higher estimator variance. Overall, POIBM with $\rho = 0.50$ appears a good compromise, with the performance generally matching that of ComBat-seq across most of the test settings.

A table of the runtimes for each of the methods, averaged over the 55 simulation settings and the 100 replicates, is shown in Table S2. The conclusions remain similar to that of the breast cancer cell line experiment: all methods can run an analysis of realistic size within minutes, the simple methods being extremely fast, followed by ComBat being an order of magnitude slower, ComBat-seq another, and POIBM yet another. Practically, POIBM also allows multithreading, which can effectively mitigate this difference effectively provided computing resources are available.

Replicate discovery in acute myeloid leukemia

As for the stomach adenocarcinoma (STAD), the TCGA dataset for acute myeloid leukemia (LAML) features a large number of overlapping samples across the processing batches. Specifically, the dataset includes 179 samples from the Illumina GA/rnaseq v1/BCGSC batch and 173 samples form the HISEQ/v2/UNC batch, with an overlap of all the 173 samples.

Regarding the POIBM estimated mapping weight 100% of the weight (173 of the 173) was on the intersecting samples, with entropy for the intersecting being ∼1.01 and for the unique ∼173 when mapping the first batch to the space of the second. For the reverse mapping, ∼98.3% (∼170 of the 173 samples) of the mapping weight was on the intersect, with the entropy for the intersecting samples being ∼1.02. This suggests that POIBM can effectively identify the replicate samples in LAML as well, regardless of the chosen mapping direction.

Supplementary figures

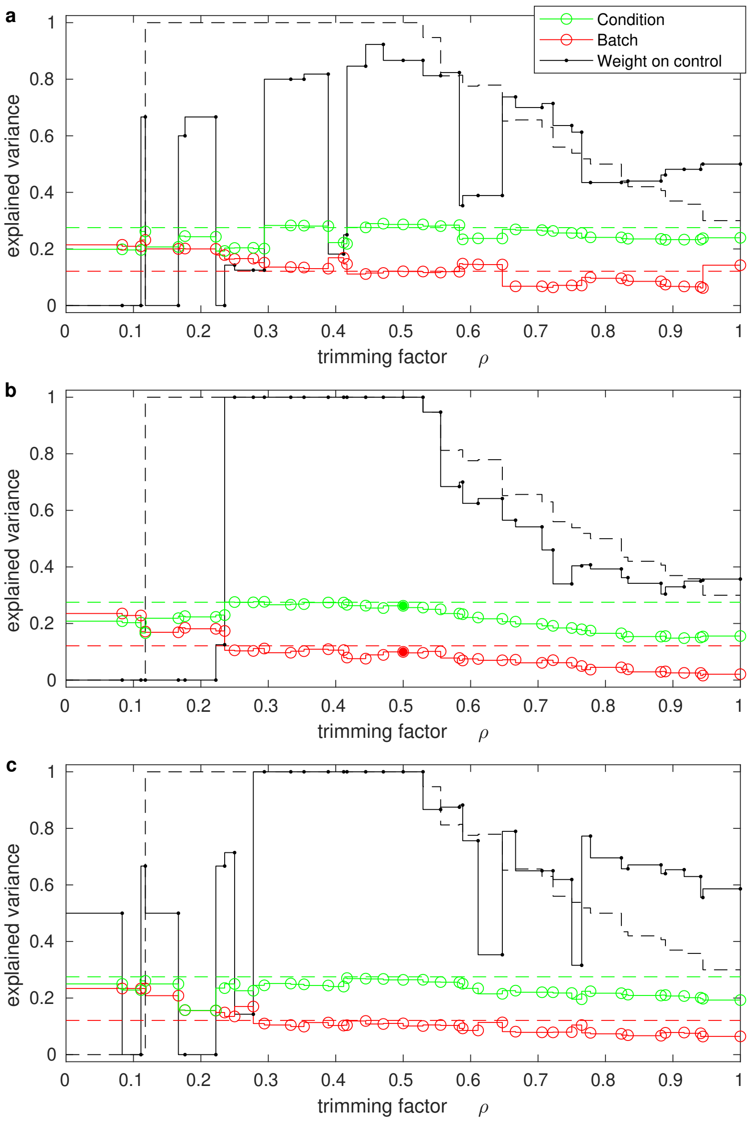

Figure S1: Effect of varying POIBM trimming factor and the target space on the explained variance on the breast cancer cell line experiment. Datasets are mapped into the space of the a) the *HER2*, b) *EGFR*, or c) *KRAS* experiment. The filled circles in b) indicate values in Table 1. The dashed lines represent the explained condition and batch variance attained by ComBat-seq and the maximum weight on control when assuming uniform weights on two or more samples.

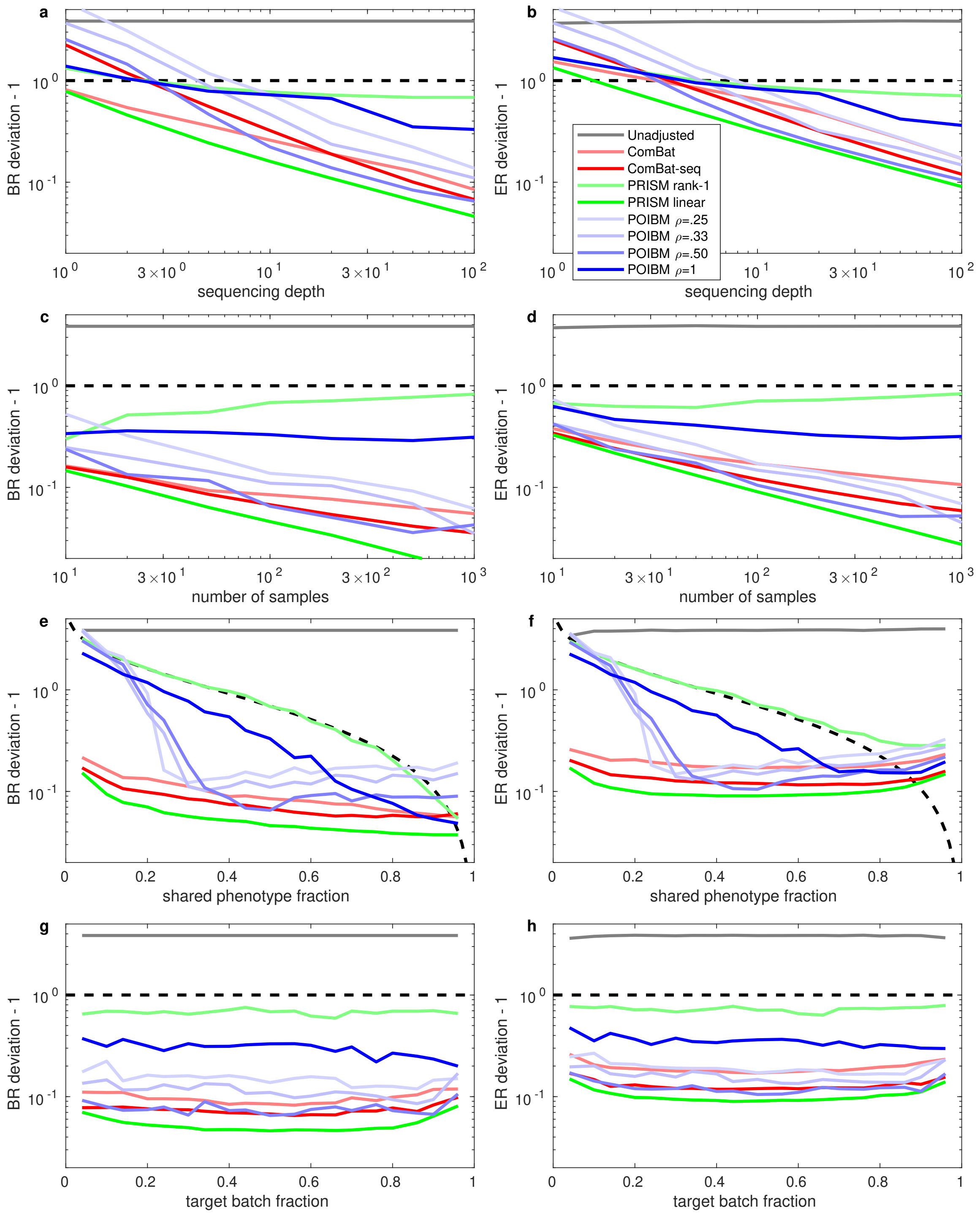

Figure S2: Parameter estimation error for various methods on simulated data. The panels show batch coefficient ratio (BR; left panels) and expression ratio (ER; right panels) deviation in excess of unity for various settings: a, b) varying sequencing depth; c, d) varying number of total samples; e, f) varying fraction of a common phenotype in the two batchs; and g, h) varying sample size fraction between the batches. The dashed lines indicate guides for a phenotype naive method. Results were averaged from 100 simulations.

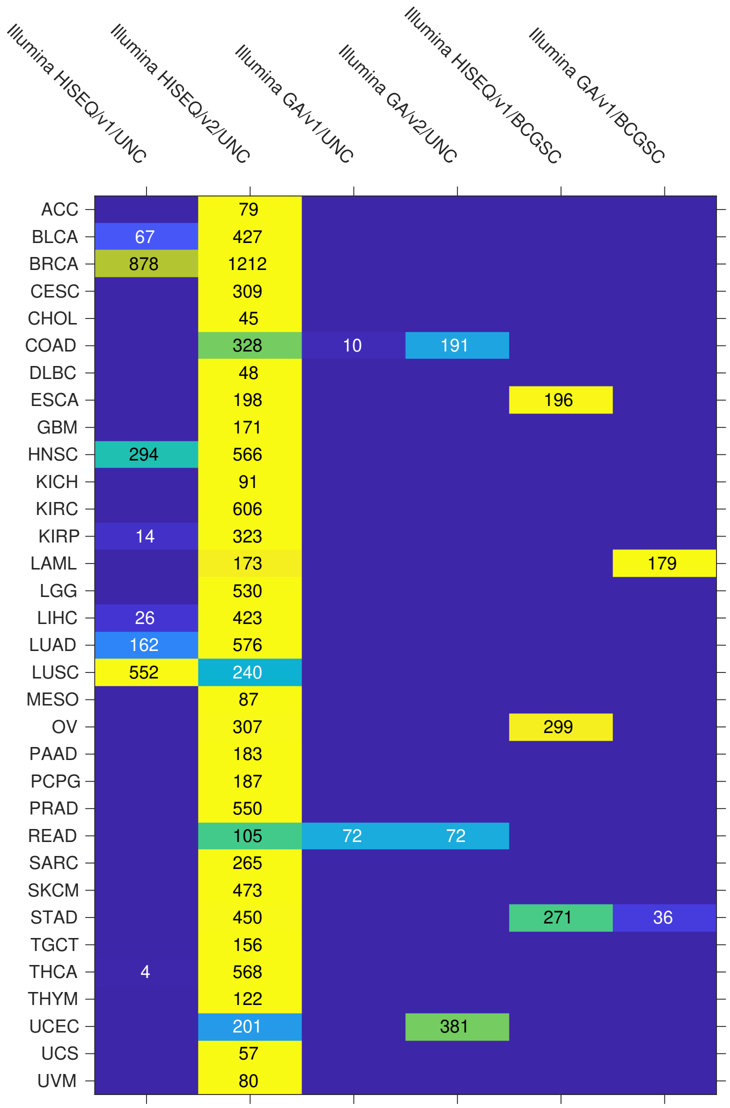

Figure S3: Distribution of samples in processing batches in TCGA data across all the cancers. Number of samples in the different processing batches in the each of the TCGA cancer types.

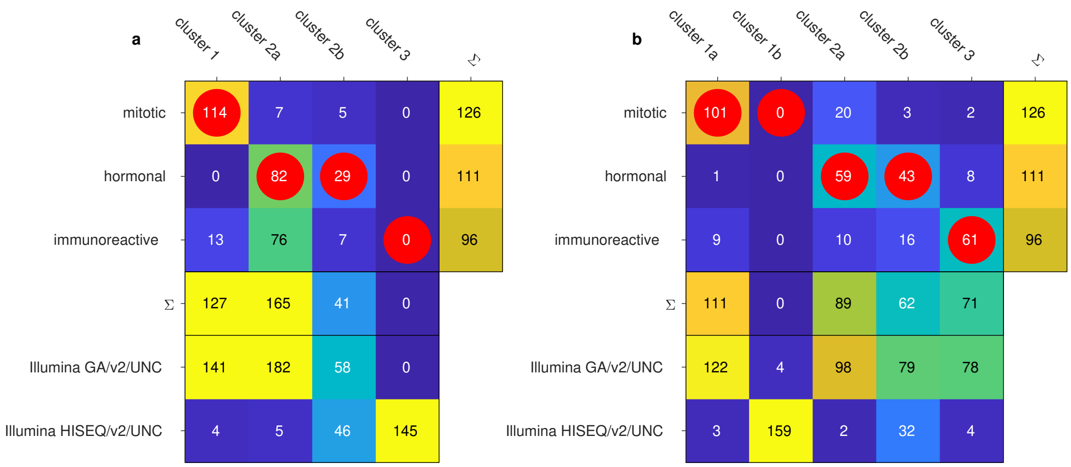

Figure S4: Reclustering of the TCGA UCEC samples. The classes "mitotic", "hormonal", and "immunoreactive" represent those from Levine *et al.* (2013), while the clusters on the columns our reproduction. Clustering of all available TCGA UCEC dataset samples, across both batches, with no batch correction, into a) four or b) five clusters. The rows and columns with Σ represent marginal sums of the original 333 samples, and the ones named after the batches all samples in each. Red circles indicate best matching clusters.

Supplementary tables

Table S1: Runtime of various methods for the breast cancer cell line experiment. The table shows mean and coefficient of variation (cv) of the expended wall clock time, the CPU time (effective time the process is scheduled), maximum CPU time over the runs, and the multithreading factor (CPU per wall clock time). The table was calculated from 100 replicate runs on an Intel Skylake system with 20× 2.4 GHz CPU cores and 88 GB of memory running Ubuntu Linux 20.04.

Method	Wall mean (s)	Wall cv	CPU mean (s)	CPU cv	CPU max (s)	Threading
ComBat	0.788	0.297	0.712	0.293	2.56	0.902
ComBat-seq	9.93	0.0371	9.87	0.0375	11.8	0.994
RUVSeq	8.48	0.0331	8.47	0.0335	9.50	0.999
PRISM rank-1	0.0224	0.106	0.0224	0.0895	0.0400	0.997
PRISM linear	0.0223	0.0986	0.0222	0.0991	0.0280	0.996
POIBM	19.9	0.0221	389	0.0185	426	19.6

Table S2: Runtime of various methods on simulated data. The table shows mean and coefficient of variation (cv) of the expended wall clock time, the CPU time (effective time the process is scheduled), maximum CPU time over the runs, and the multithreading factor (CPU per wall clock time). The table was calculated from 100 replicate runs averaged over the 55 simulation settings on an Intel Skylake system with 20× 2.4 GHz CPU cores and 88 GB of memory running Ubuntu Linux 20.04.

Method	Wall mean (s)	Wall cv	CPU mean (s)	CPU cv	CPU max (s)	Threading
Unadjusted	0.000323	0.835	0.000323	0.847	0.00100	0.996
ComBat	0.649	0.0621	0.574	0.0483	0.640	0.884
ComBat-seq	13.2	0.0356	13.0	0.0354	14.2	0.985
PRISM rank-1	0.0318	0.0931	0.0278	0.0694	0.0321	0.870
PRISM linear	0.0253	0.0751	0.0226	0.0612	0.0315	0.892
POIBM $\rho = .25$	1.56	0.415	25.7	0.365	64.7	16.2
POIBM $\rho = .33$	3.19	0.387	52.7	0.329	117	16.5
POIBM $\rho = .50$	3.24	0.406	55.3	0.384	131	16.9
POIBM $\rho = 1$	2.43	0.500	41.4	0.488	112	16.9
